# Supplementary material for: Safety and Feasibility of Neoadjuvant-Modified FOLFIRINOX in Elderly Patients with Pancreatic Cancer
Source: Cancers (Basel). 2024 Jul 12;16(14):2522. doi: 10.3390/cancers16142522 (PMC11275028; doi:10.3390/cancers16142522)
Supplement: Supplementary file 1 [file cancers-16-02522-s001.zip › cancers-3061180-supplementary.pdf]

Supplemental Table S1. Surgical outcomes of patients who underwent pancreaticoduodenectomy.

	**Age≥75 years (n=9)**	**Age<75 years (n=18)**	***P*-value**
Operation time (min)	561 (430–850)	581 (465–805)	0.7814
Blood loss (ml)	500 (130–2800)	643 (200–4450)	0.4948
Clavien-Dindo ≥IIIA	5 (56)	11 (61)	>0.9999
POPF of ≥grade B	0 (0)	3 (17)	0.5292
DGE of ≥grade B	0 (0)	1 (6)	>0.9999
Postoperative hospital stay (days)	19 (15–79)	25 (10–70)	0.3406

POPF, Postoperative pancreatic fistula; DGE, delayed gastric emptying.

Supplemental Table S2. Surgical outcomes of patients who underwent distal pancreatectomy.

	**Age≥75 years (n=10)**	**Age<75 years (n=10)**	***P*-value**
Operation time (min)	374 (280–532)	387 (273–502)	0.9705
Blood loss (ml)	269 (25–1380)	200 (10–360)	0.5923
Clavien-Dindo ≥IIIA	2 (20)	5 (50)	0.3498
POPF of ≥grade B	3 (30)	3 (30)	>0.9999
DGE of ≥grade B	0 (0)	1 (10)	>0.9999
Postoperative hospital stay (days)	17 (10–40)	17 (13–60)	0.8670

POPF, Postoperative pancreatic fistula; DGE, delayed gastric emptying.

Supplemental Table S3. Postoperative complications stratified by G8 score.

	**G8>14 (n=15)**	**G8≤14 (n=34)**	***P*-value**
Clavien-Dindo ≥IIIA	8 (53)	15 (44)	0.7569
Any Morbidity	10 (67)	20 (59)	0.7536
Biliary leakage	1 (7)	1 (3)	0.5230
POPF of ≥grade B	2 (13)	7 (21)	0.7021
DGE of ≥grade B	2 (13)	1 (3)	0.2185
Intraabdominal bleeding	0 (0)	1 (3)	>0.9999
Gastrointestinal bleeding	1 (7)	0 (0)	0.3061
Intraabdominal abscess	4 (27)	8 (24)	>0.9999
Chylous ascites	2 (13)	0 (0)	0.0893
Cholangitis	0 (0)	1 (3)	>0.9999
Pneumoniae	0 (0)	1 (3)	>0.9999
Delirium	0 (0)	1 (3)	>0.9999
Heart failure	0 (0)	1 (3)	>0.9999
Renal failure	0 (0)	1 (3)	>0.9999

POPF, Postoperative pancreatic fistula; DGE, delayed gastric emptying.
